# Diversity and distribution of the eukaryotic picoplankton in the oxygen minimum zone of the tropical Mexican Pacific

**DOI:** 10.1093/plankt/fbae083

**Published:** 2025-03-01

**Authors:** David U Hernández-Becerril, Raquel Rodríguez-Martínez, Francisco Varona-Cordero, Martín Merino-Ibarra, Píndaro Díaz-Jaimes, Silvia Pajares

**Affiliations:** Instituto de Ciencias del Mar y Limnología, Universidad Nacional Autónoma de México (UNAM), Ciudad Universitaria, Coyoacán, PC: 04510, Ciudad de México, Mexico; Laboratorio de Complejidad Microbiana y Ecología Funcional, Instituto Antofagasta, Universidad de Antofagasta, Angamos 601, PC: 1240000, Antofagasta, Chile; Departamento de Biotecnología, Facultad de Ciencias del Mar y Recursos Biológicos, Universidad de Antofagasta, Angamos 601, PC: 1240000, Antofagasta, Chile; Centre for Biotechnology and Bioengineering (CeBiB), Angamos 601, PC: 1240000, Santiago, Chile; Instituto de Ciencias del Mar y Limnología, Universidad Nacional Autónoma de México (UNAM), Ciudad Universitaria, Coyoacán, PC: 04510, Ciudad de México, Mexico; Instituto de Ciencias del Mar y Limnología, Universidad Nacional Autónoma de México (UNAM), Ciudad Universitaria, Coyoacán, PC: 04510, Ciudad de México, Mexico; Instituto de Ciencias del Mar y Limnología, Universidad Nacional Autónoma de México (UNAM), Ciudad Universitaria, Coyoacán, PC: 04510, Ciudad de México, Mexico; Instituto de Ciencias del Mar y Limnología, Universidad Nacional Autónoma de México (UNAM), Ciudad Universitaria, Coyoacán, PC: 04510, Ciudad de México, Mexico

**Keywords:** picoeukaryotes, oxygen minimum zone, tropical Mexican Pacific, metabarcoding, flow cytometry, distribution patterns

## Abstract

The ecology of eukaryotic picoplankton in oxygen minimum zones (OMZs) is crucial to understand global primary production, trophic dynamics and plankton diversity. This study analyses picoeukaryotic diversity and distribution patterns along the water column at two locations (slope and oceanic) in the tropical Mexican Pacific OMZ using metabarcoding and flow cytometry. Well-known groups of Chlorophytes (Mamiellophyceae) and Ochrophytes (Chrysophyceae, Dictyochophyceae, Pelagophyceae) occurred in high relative abundances, whereas less-known groups such as Chloropicophyceae and Prasinodermophyta were found in lower abundances. Picoeukaryotic diversity was higher at the lower end of the oxycline (10 μM O_2_) than at the surface and subsurface layers. Differential distributions of picoeukaryotes were also detected along the water column, with almost exclusive communities at each depth. Mamiellophyceae dominated the surface and subsurface layers, whereas Syndiniales (parasitic dinoflagellates), Radiolaria, Ochrophyta, and Sagenista (MArine STramenopiles -MAST groups-) were prevalent at the oxycline. Post-upwelling oceanographic conditions possibly contributed to shape the differences in community composition and distribution. These findings highlight that oxygen concentration is a key factor driving microbial distribution and that oxyclines provide specialized niches that promote high picoplankton diversity and multiple trophic strategies including autotrophy, mixotrophy, heterotrophy and parasitism.

## INTRODUCTION

The diversity and distribution of marine microorganisms is an important research topic to understand the primary productivity and trophic dynamics of the plankton. Marine picoplankton (<3 μm size fraction) is composed of both prokaryotic and eukaryotic microorganisms that can be autotrophs (photosynthetic forms), mixotrophs and heterotrophs. They play important ecological roles as primary producers, predators or symbionts and dominate marine ecosystems in terms of cell densities and carbon biomass (Li, 1994; Raven, 1998; Worden *et al*., 2004; Buitenhuis *et al*., 2012). The well-known cyanobacteria genera *Synechococcus* and *Prochlorococcus* are the main prokaryotic components of the photosynthetic picoplankton, whereas the eukaryotic community includes several taxonomic groups such as Chlorophyta (Chloropicophyceae, Mamiellophyceae), Ochrophyta (Bolidophyceae, Bacillariophyceae, Chrysophyceae, Pelagophyceae) and Haptophyta (Massana and Pedrós-Alió, 2008; Vaulot *et al*., 2008; Worden and Not, 2008; Massana, 2011; Buitenhuis *et al*., 2012; Yuan *et al*., 2021). The diversity of this community has been studied traditionally by microscopic techniques and, more recently, by molecular tools such as metabarcoding (Andersson *et al*., 2023). This approach has become a useful method for assessing marine microbial diversity (Moon-van der Staay *et al*., 2001; Massana and Pedrós-Alió, 2008; de Vargas *et al*., 2015; Yuan *et al*., 2021), being especially relevant in numerous discoveries of major lineages of mostly free-living heterotrophic protists (Burki *et al*., 2021).

Oxygen minimum zones (OMZs) are certain oceanic areas that have a very low concentration of dissolved oxygen (<20 μM) very close to surface (Paulmier and Ruiz-Pino, 2009). It was recently predicted that OMZs are going to spread in the coming decades as a consequence of global climate change (Oschlies, 2021). OMZs are also keys to understand the current unbalanced in the global nitrogen cycle and the role of the oceans on atmospheric greenhouse control (Lam and Kuypers, 2011; Bianchi *et al*., 2012). The paucity of oxygen in OMZ supposes great challenges to marine life, especially to the microbial communities. These communities are dominated by prokaryotes that can develop several strategies, including oxygenic and anoxygenic photosynthesis, as well as aerobic, anaerobic and microaerophilic metabolisms (Ulloa *et al*., 2012; Wright *et al*., 2012; Sunagawa et al., 2015).

The world’s largest permanent OMZ area is in the Eastern Tropical North Pacific (ETNP), which is a tropical and subtropical region occupied by most of the Mexican Pacific (Paulmier and Ruiz-Pino, 2009; Maske *et al*., 2019). Although the relative high diversity of small-sized microorganisms (including picoplankton) in some OMZ areas is already known (Orsi *et al*., 2012; Parris *et al*., 2014; Duret *et al*., 2015; Jing *et al*., 2015; Bertagnolli and Stewart, 2018; de la Iglesia *et al*., 2020; Giner *et al*., 2020; Suter *et al*., 2022), the diversity, abundance and distribution of the picoeukaryotic community have been only recently studied in the Mexican Pacific OMZ (Santana-Vega *et al*., 2018; Pajares *et al*., 2020; Hernández-Becerril *et al*., 2024). The formation of a second peak of deep chlorophyll *a* maximum in these areas, especially in remote oceanic sites (Cepeda-Morales *et al*., 2009; Márquez-Artavia *et al*., 2019), has been confirmed in close association to high abundances of the picocyanobacterium *Prochlorococcus* (Goericke *et al*., 2000; Santana-Vega *et al*., 2018; Pajares *et al*., 2020; Hernández-Becerril *et al*., 2024).

Other studies of pico-nanoplankton eukaryotic communities in the Mexican Pacific OMZ showed that Alveolata and Rhizaria dominated two size fractions (>30 and 1.6–30 μm), with the community composition at finer taxonomic levels partially shaped by oxygen concentrations (Duret *et al*., 2015). Syndiniales Group I, dinoflagellate endoparasites from Alveolata, was found in the 1.6–30 μm fraction with high proportions at the OMZ core (800 m depth) and lower oxycline (1000 m depth), likely due to microniches on sinking particles (Duret *et al*., 2015; Bertagnolli and Stewart, 2018). Additionally, a vertical distribution study of planktonic groups (not necessarily picoplanktonic) in the Costa Rica OMZ revealed diatoms dominating surface waters, with a clear shift from photosynthetic dinoflagellates in surface waters to parasitic dinoflagellates and ciliates in deeper waters. The low community diversity and dominance of parasitic groups in the anoxic core suggest selective pressure on protist communities (Jing *et al*., 2015).

Furthermore, in the OMZ off northern Chile, picoeukaryotic (0.2–1.6 μm) diversity decreased from a maximum at the OMZ core to the lower oxycline (Parris *et al*., 2014). Dinophyceae and Syndiniales dominated the protist assemblage across depths within the OMZ, showing depth-specific variations despite uniform oxygen conditions (Parris *et al*., 2014). de la Iglesia *et al*. (2020) further highlighted the dominance of these groups and their temporal dynamics in the OMZ off central Chile driven by stratification changes in the water column.

The present study is based on samples collected at several depths along the water column at two locations in the tropical Mexican Pacific OMZ. An approach of metabarcoding and flow cytometry was used to assess the presence and abundance of taxonomic groups of picoeukaryotes, as well as to determine their diversity and distribution patterns in this OMZ. We anticipated highly diverse picoeukaryotic communities, given the high diversity of several taxonomic groups within the nano- and microplankton communities (dinoflagellates, diatoms, coccolithophores and silicoflagellates, among others) previously reported in the same area by microscopic studies (Hernández-Becerril *et al*., 2021). We expected these communities to exhibit differential distributions driven by oceanographic and environmental conditions, particularly dissolved oxygen concentrations.

## MATERIALS AND METHODS

### Sample collection, physicochemical profiles and chemical analyses

**Fig. 1:**
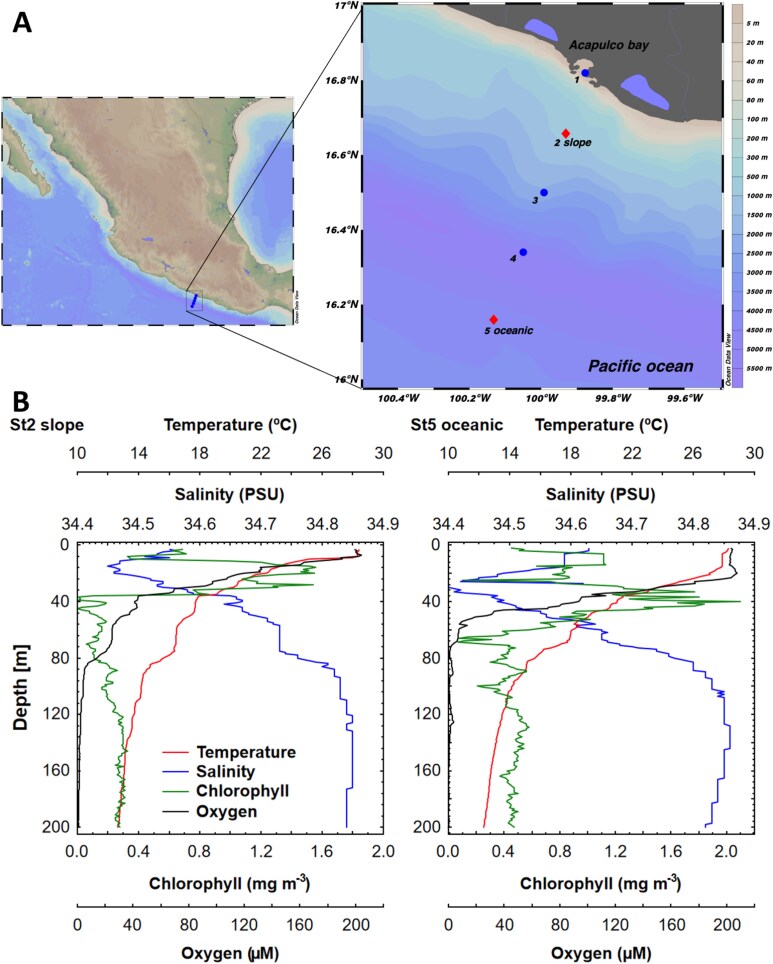
(**A**) Study region in the Tropical Mexican Pacific showing the locations of the two sampling stations. (**B**) Vertical profiles of physico-chemical variables (dissolved oxygen, chlorophyll-a, salinity and temperature) in the two sampling stations.

The study area is located off the Mexican Pacific coast, within the OMZ of the ETNP (Fig. 1). Hydrographic data and seawater samples were collected along a cross-shore transect of five stations in front of Acapulco during the oceanographic cruise “MareaR-X” on board the R/V “El Puma” (UNAM). Sampling was conducted in April 2018, coinciding with a moderate La Niña, which is the cold phase of the inter-annual climatic variability of El Niño Southern Oscillation (multivariate ENSO index: −1.3, data from https://psl.noaa.gov/enso/mei/).

Temperature, dissolved oxygen, salinity and fluorescence (chlorophyll *a*) profiles were measured with an SBE-19plus CTD probe equipped with a WET labs fluorometer sensor. Seawater samples for molecular analyses of picoplankton were collected from a slope station (St2: 767 m depth) and an oceanic station (St5: 4420 m depth) in 10 L Niskin bottles deployed on the CTD rosette at different depths based on both oxygen and chlorophyll *a* profiles: surface (10 m depth), deep chlorophyll maximum (DCM, 20 m at the slope station and 40 m depth at the oceanic station), lower limit of the DCM at the slope station (30 m depth) and the lower end of the oxycline (~10 μM O_2_, 81 m depth at the slope station and 62 m depth at the oceanic station). In addition, samples for nutrient (in the five stations), picoplankton abundance analyses and microscopical studies were collected at specific depths from the surface to the lower end of the oxycline. For microscopical studies, 3–4 L of the sample were filtered through 5 μm cellulose filters; then, the filters were rinsed with distilled water, air-dried and kept for future observations.

Duplicated seawater samples for nutrient analyses were filtered on board through cellulose acetate syringe filters (0.22 μm pore, Merck Millipore), collected in acid-washed polypropylene containers and stored at −20°C until their analysis. Analyses of NH_4_^+^, NO_2_^−^, NO_3_^−^, PO_4_^−3^ and SiO_2_ were determined using a Skalar San-Plus segmented-flow autoanalyser (Hansen and Koroleff, 1999). The detection limits for these analyses were as follows: 0.1 μM for NH_4_^+^, 0.02 μM for NO_2_^−^, 0.1 μM for NO_3_^−^, 0.04 μM for PO_4_^−3^ and 0.1 μM for SiO_2_.

### Microscopy and picoplankton abundance analyses

Filters with phytoplankton were studied by scanning electron microscopy (SEM). Pieces of filters were mounted on aluminium stubs, coated with gold and observed directly by SEM (JEOL JSM6360LV).

For picoplankton abundance analysis, 5 mL of water of the selected depths were fixed with 1% glutaraldehyde and frozen in liquid nitrogen. In the laboratory, samples were thawed at room temperature and then injected into a FACSCalibur (Becton Dickinson) flow cytometer (Marie *et al*., 2001). The following parameters were considered: forward light scatter (FSC, E01), side light scatter (SSC, 450) and four fluorescence (FL1, 650 green, FL2, 650 orange, FL3, 650 red and FL4, 650 red). Fluorescence microspheres of 2 μm (Becton Dickinson) were used for calibration. Picophytoplankton populations were identified and quantified by analysing the cytograms with the software Cyflogic 1.2.1 (Cyflo-LTD 2008) (Fig. S1). Picoplankton biomass (μg C L^−1^) was calculated based on the picoplankton abundances and using the factors corresponding to each group: 56, 112 and 1 010 fg cell L^−1^ for *Prochlorococcus*, *Synechococcus* and picoeukaryotes, respectively (Lee and Fuhrman, 1987; Durand *et al*., 2001; Linacre *et al*., 2010).

### Picoplankton DNA extraction, sequencing and amplicon data processing

Triplicate 1.3 L seawater samples for DNA were pre-filtered through 3 μm polycarbonate membranes and then filtered through 0.22 μm polycarbonate membranes (Millipore). Filters of 0.22 μm pore size, containing the 0.2 to 3 μm size fraction, were stored at −80°C until DNA analysis.

Genomic DNA was extracted from three filters per depth with the DNeasy PowerWater Kit (Qiagen), according to the manufacturer’s instructions, and then pooled at equal volumes. DNA samples were shipped to the Massive Sequencing and Bioinformatics Unit of the Institute of Biotechnology (UNAM) for PCR amplification and Illumina MiSeq sequencing (2 × 300 bp) of the 18S rRNA gene V4 region with the index and adaptor-linked primers V4_18SNext.For (5′- CCA GCA SCY GCG GTA ATT CC) and V4_18SNext.Rev (5′- ACT TTC GTT CTT GAT YRA TGA) (Piredda *et al*., 2017). Two sequencing runs per DNA sample were performed to ensure good sequencing performance.

Amplicon libraries were analysed using the DADA2 package (Callahan *et al*., 2016) version (1.16) in “R” 4.1.0 (R Core Team, 2021). After inspection of read quality profiles, the primers were removed, and the forward reads were trimmed to 240 bases and the reverse ones to 210 bases. The maximum number of expected errors (maxEE) was set to 2 for both forward and reverse reads. The two runs were analysed separately and pooled with the “mergeSequenceTables” function. The chimera sequences were excluded after merging the different runs using the function “removeBimeraDenovo.” The taxonomic assignment of the ﻿amplicon sequence variants (ASVs) was performed using the PR2 V4.14.0 as a reference database (Guillou *et al*., 2013). Only sequences assigned as eukaryote were kept and ASVs assigned to Metazoa were removed.

### Data analyses

A temperature-salinity (T-S) diagram was made to identify water masses within the study area using conservative temperature and absolute salinity calculated as derived variables in Ocean Data View v. 5.0 (Schlitzer, 2015). The Pacific Coastal Upwelling Index (CUI, or Bakun Index) was used as a measure of coastal upwelling conditions during the cruise and was obtained for the location 21° N, −107° W from the National Marine Fisheries Service Environmental Lab (http://www.pfeg.noaa.gov). The environmental data describing the oceanographic conditions in the studied area were sea surface temperature (SST), diffuse attenuation coefficient at 490 nm (Kd490) and surface chlorophyll *a* concentration (Chla), which were obtained from the Moderate Resolution Imaging Spectroradiometer (MODIS) from NASA’s Aqua satellite (https://coastwatch.pfeg.noaa.gov/erddap/index.html).

A stacked bar chart was generated with the reshape2 v. 1.4.4 and ggvis v. 0.4.7 packages in R to identify the relative abundance of the taxonomic composition of picoeukaryotes classified by “Division” in the samples. A heatmap showing the abundance distribution by sample of taxonomic groups classified up to the “Genus” level contributing at least 0.5% was generated using the Euclidean dissimilarity distance and the complete hierarchical clustering algorithm with the pheatmap package v. 1.0.12 (Kolde, 2015) in R.

The abundance table was rarefied (138 000 reads per sample) using the rarefy function with the vegan package v. 2.5-7 (Oksanen *et al*., 2020) in R to correct the uneven sequencing depths among samples in order to calculate alpha diversity indices (Chao1, Shannon, Simpson) and beta diversity analysis (Bray–Curtis distance matrix). Differences in beta diversity between stations and depths were tested through permutational multivariate analysis of variance (PERMANOVA) and analysis of similarities (ANOSIM), using the adonis2- and anosim functions from vegan, to evaluate whether community structures differed significantly across sampling locations and depths. A principal coordinates analysis (PCoA) based on the Bray–Curtis distance matrix was conducted with vegan to assess community dissimilarity among samples and identify clustering patterns related to environmental gradients. The envfit function in vegan was used to fit significant environmental vectors (*P* < 0.1) to the plot, assessing the influence of environmental factors on community structure. The PCoA plot was generated with the ggplot2 package ﻿v. 3.3.5 ﻿(Wickham *et al*., 2021). To explore the overlap and uniqueness of ASVs and taxa at the genus level across stations and depths, Venn diagrams were created with the ggVennDiagram package v. 1.2.2 (Gao *et al*., 2021). These diagrams provide insights into community partitioning across samples.

## RESULTS

### Hydrographic and oceanographic conditions

Based on the criteria established by Portela *et al*. (2016), the T-S diagram showed the presence of four water masses (from the surface down to 1200 m depth at the most offshore stations) in the study area (Fig. S2): (i) ﻿tropical surface water (TSW), characterized by oxygenated surface waters (>200 μM), temperature > 25.1°C and absolute salinity <34.6 g kg^−1^, which was only present at the stations St2 and St3; (ii) transitional water (TrW), down to 20–40 m depth and derived from the mixture of TSW and the subtropical subsurface water (StSsW); (iii) StSsW, intermediate waters with low oxygen levels (<9 μM), temperature ranging from 9 to 18°C and absolute salinity between 34.6 and 35 g kg^−1^, ﻿which extended to 440–460 m depth (at all stations, except the St1); ﻿ (iv) and Pacific Intermediate water (PIW), anoxic waters with temperature ranging from 4 to 9°C and absolute salinity between 34.6 and 34.9 g kg^−1^, ﻿which was below the StSsW mass.

According to the CUI index, upwelling events (>100 m^3^/s × 100 m) took place during April 2018 (Fig. S3), with the highest intensities reaching 175 and 226 m^3^/s × 100 m on 4 and 18 April 2018, respectively. During the cruise, SST varied from 28.5°C at the coastal station St1 to 30°C at the oceanic station St5 (Fig. S4a). SST was lower in the coast because of the intrusion of the StSsW mass and the consequent displacement of the TSW mass towards the open ocean. The diffuse attenuation coefficient Kd490 decreased from the bay (St1: 0.07 m^−1^) to the open ocean (St5: <0.05 m^−1^) (Fig. S4b). The surface chlorophyll *a* concentration followed a similar trend, with the highest values occurring at the coastal station St1 (2.0 mg m^−3^) and the lowest values at the oceanic station St5 (<0.2 mg m^−3^), representative of more oligotrophic waters (Fig. S4c).

Hydrographic variables measured *in situ* along the transect off Acapulco (from the coastal station St1 to the most remote station St5) showed evident gradients with notable differences between the two stations where picoplankton samples were studied (St2 and St5) (Figs 1 and S5). Temperature showed a warm layer (>25.1°C) from the surface to 10 m depth at the coastal station St1 and to 25 m depth in the oceanic station St5. The thermocline at the slope station St2 (between 10 and 35 m depth) was slightly shallower than that at the oceanic station St5 (between 25 and 40 m depth). The DCM layer close to the coast (16–20 m depth at St2) was much shallower than in the open ocean (~75 and 40 m depth at St4 and St5, respectively). An intense second DCM was only found at ~90 m depth at the oceanic station St4 (Fig. S5). The dissolved oxygen profiles followed the classical OMZ pattern, decreasing sharply (oxycline) from 10 to 81 m depth at St2 and from 30 to 62 m depth at St5. The upper limit of the OMZ (9 μM O_2_) was shallower (40–50 m depth) at the intermediate stations of the transect (St3 and St4). A lower salinity plume (<34.6) was identified across the transect, which deepened towards the open ocean (from 30 to 50 m depth) because of the intrusion of epicontinental water from Acapulco Bay.

**Fig. 2:**
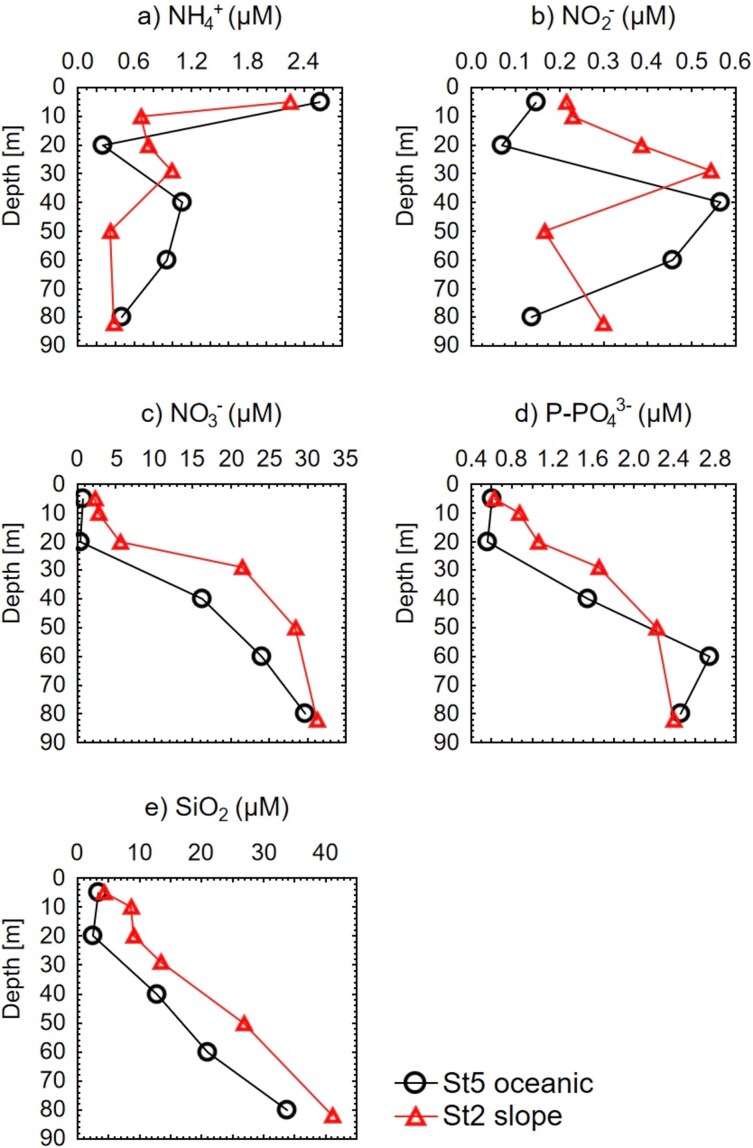
Vertical profiles of nutrient concentrations in the two sampling stations: (**a**) ammonium (NH_4_^+^), (**b**) nitrite (NO_2_^−^), (**c**) nitrate (NO_3_^−^), (**d**) soluble reactive phosphorus (PO_4_^−3^) and (**e**) soluble reactive silica (SiO_2_). The circles indicate the sampling depths (mean values obtained from two replicate samples per depth).

Nutrient concentrations in the surface layer, especially NO_3_^−^, PO_4_^−3^ and SiO_2_, were slightly higher at the most coastal station St1 than at the most oceanic station St5, and they showed a typical increase from the surface to deeper layers across all stations, except at St1 due to its proximity to the coast (Figs 2 and S6). In contrast, NH_4_^+^ and NO_2_^−^ followed different patterns from the other nitrogen species, with NH_4_^+^ having the highest concentrations at the surface, whereas NO_2_^−^ exhibited its characteristic maximum associated with the oxycline, observed at 30 and 40 m depths at St2 and St5, respectively (Fig. 2).

### Picoplankton abundances

**Fig. 3:**
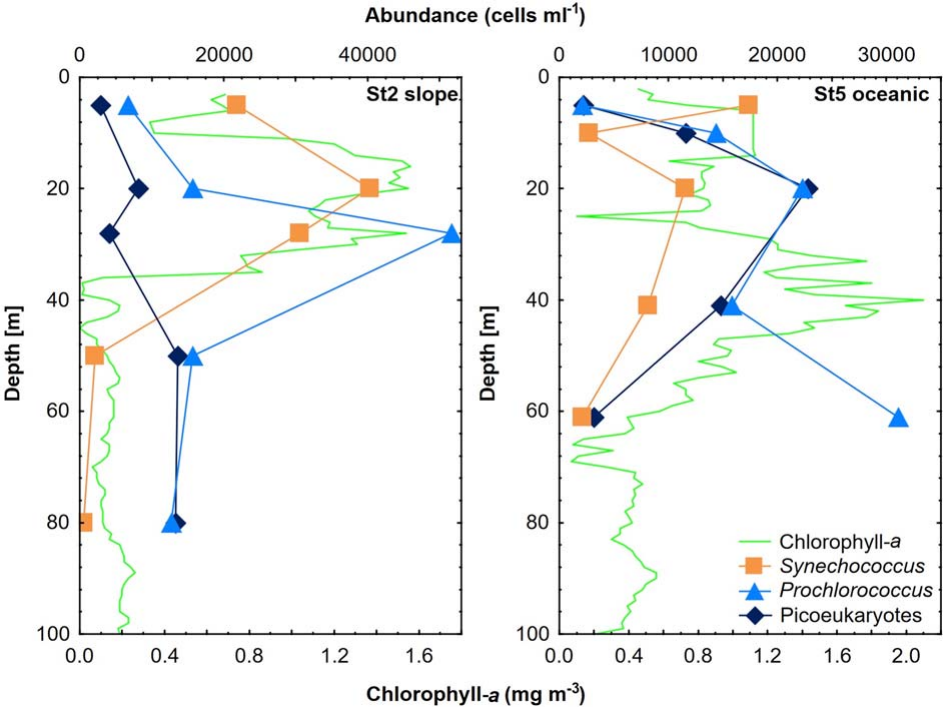
Vertical profiles of the abundance of picoplankton detected by flow cytometry in the two sampling stations.

The abundances of both prokaryotic and eukaryotic picoplankton communities were assessed at the stations St2 and St5 (Figs 3 and S1). *Synechococcus* was the most abundant picoplankton at the surface of both stations, with 2.2 × 10^4^ cells mL^−1^ at St2 and 1.74 × 10^4^ cells ml^−1^ at St5, reaching a peak of 4.0 × 10^4^ cells mL^−1^ at the DCM layer (20 m depth) of St2. *Prochlorococcus* showed its highest abundance at the lower limit of the DCM at St2 (5.2 × 10^4^ cells mL^−1^ at 30 m depth), whereas it had a peak above the DCM layer at St5 (2.2 × 10^4^ cells ml^−1^ at 20 m depth), reaching the highest abundance at the lower end of the oxycline (3.1 × 10^4^ cells ml^−1^ at 62 m depth). Eukaryotic picoplankton was more abundant at St5 than at St2, showing a clear maximum above the DCM at St5 (2.3 × 10^4^ cells mL^−1^ at 20 m depth), whereas it had a more homogeneous vertical distribution at St2 (0.3–1.3 × 10^4^ cells mL^−1^).

Eukaryotic picoplankton contributed with 67.7% of the total picophytoplankton biomass (8.5 μg C l^−1^), whereas *Synechococcus* and *Prochlorococcus* contributed with 22.1% (2.14 μg C l^−1^) and 10.2% (1.15 μg C l^−1^), respectively.

### Taxonomic composition and distribution of the eukaryotic picoplankton

﻿Illumina sequencing generated 2100 ASVs in 2 026 475 ﻿quality-filtered reads, ﻿ranging from 138 761 to 494 953 reads per sample (Table S1). As expected, the percentage of removed reads belonging to Metazoa was very low (3.9%), given that the analyzed fraction is picoeukaryotic and the primers used do not target Metazoa. Molecular analyses showed a high complexity of taxonomic groups belonging to the picoeukaryotic community throughout the water column of both sampled stations, with 24.4 and 12.8% of the total ASVs assigned to known genus and species, respectively (Table S1).

**Fig. 4:**
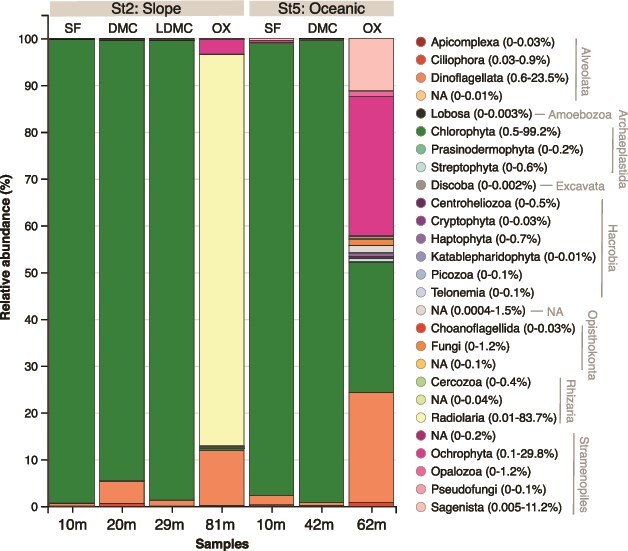
Comparison of the relative abundance of microbial eukaryotic groups classified by “Division.” The two sampling stations are represented at different depths: SF, DCM, LDCM and OX. The percentage range of every clade in the samples is indicated in parentheses. NA: uncharacterized phylogenetic groups that branch with the assigned taxon in the PR2 database classification.

At the Division level, the most abundant groups were Chlorophyta (up to 99.2%), Radiolaria (up to 83.7%), Ochrophyta (up to 29.8%), Dinoflagellata (up to 23.5%) and Sagenista (up to 11.2%) (Fig. 4). Photosynthetic groups such as Chlorophyta clearly dominated the surface and subsurface layers (including DMC and LDMC depths). In contrast, at the lower end of the oxycline, Radiolaria (heterotrophs) were abundant at the slope station St2, whereas Ochrophyta (mostly photosynthetic but also mixotrophic and heterotroph forms), Dinoflagellata and Sagenista (mostly heterotrophs) increased at the oceanic station St5. Additionally, there was a notable presence of Fungi (up to 1.2%) and Opalozoa (up to 1.2%) at this layer of the St5.

**Fig. 5:**
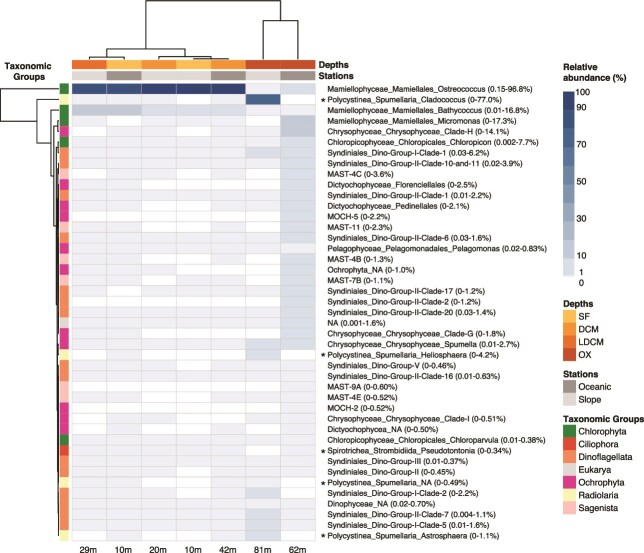
Heatmap showing the distribution by sample of the most abundant groups (>0.5%) classified up to genus level assigned in the PR2 database. The colour key scale shows the percentage range of the relative abundance (white colour denotes no presence of the group). Rows represent clades (the percentage range in the samples is indicated in parentheses), and columns represent the samples. Dendrograms show hierarchical clustering among samples and clades, respectively. Labels of the sampling depths: SF, surface; DCM, deep chlorophyll maximum; LDCM, lower limit of the DCM in the slope station; OX, end of the oxycline. Asterisks indicate microorganisms associated to a size bigger than picoplankton fraction.

Within Chlorophyta, Mamiellophyceae showed a high relative abundance, with *Ostreococcus* (basically clade B, 100% Blast identity) and *Bathycoccus* (mainly *Bathycoccus prasinos*, 100% Blast identity) as the most abundant genera (up to 96.8% and 16.8%, respectively) at the surface and subsurface layers of both stations, whereas *Micromonas* (mainly *Micromonas commoda*, 99.2% Blast identity) was the most abundant (17.3%) at the lower end of the oxycline of St5 (Fig. 5, Tables S1 and S2). A remarkable finding in all the samples was the presence of little-known or relative recently described groups of Chloropicophyceae, such as *Chloropicon* and *Chloroparvula* (Fig. 5). *Chloropicon* (up to 7.71%) was abundant at the lower end of the oxycline of St5 and was mostly represented by *Chloropicon roscoffensis* (7.65%, 100% Blast identity). *Chloroparvula* (up to 0.38%) was represented mainly by *Chloroparvula pacifica* (98.94% Blast identity) and had low abundances with a homogeneous distribution along the water column of both stations. Also, the chlorophyte-like taxonomic group Prasinodermophyta, basically represented by *Prasinoderma coloniale* (99.73% Blast identity), was found in low abundance (0.16%) at the lower end of the oxycline of St5 (Tables S1 and S2).

Ochrophyta was another relevant taxonomic group represented by diverse classes, particularly at the lower end of the oxycline of St5 (Figs 4 and 5). Notably, several Chrysophyceae clades were detected, such as the heterotroph *Spumella* (up to 2.7%; mainly at both oxyclines), as well as Clade H (14.1%) and Clade G (1.8%), while Dictyochophyceae were mainly represented by Florenciellales (2.5%) and Pedinellales (2.1%). Bolidophyceae were identified through parmales (0.45%), including *Triparma eleuthera* and *T. pacifica* (100% Blast identity), whereas *T. laevis* was only observed by SEM (Fig. S7). Clades MOCH-5 (2.2%) and MOCH-2 (0.52%) were also detected at the oxycline of St5. Additionally, Pelagophyceae were distributed throughout the water column and primarily represented by *Pelagomonas calceolata* (up to 0.83%, 100% Blast identity). Bacillariophyceae (diatoms) were less abundant (<0.1%) than other related groups, but exhibited a high diversity of taxa, particularly at the slope station St2. Notable examples include small-sized species such as *Thalassiosira oceanica* (96.3% Blast identity, also detected by SEM), *Chaetoceros tenuissimus* (100% Blast identity) and *Minidiscus* spp. (100% Blast identity, also observed by SEM) (Fig. S7, Tables S1 and S2).

Other photosynthetic groups were also found in low relative abundances, mainly at the oxycline of St5. These included Prymnesiophyceae (0.72%; Haptophyta), mostly *Chrysochromulina* and *Phaeocystis*, and Chlorarachniophyceae (0.34%; Cercozoa), with species reported for the first time in the Mexican Pacific, such as *Partenskyella glossopodia* (100% BLAST identity) and its only known heterotrophic species *Minorisa minuta* (100% BLAST identity) (Tables S1 and S2).

Numerous taxa of Dinoflagellata belonging to Dinophyceae and Syndiniales were found in the samples (Fig. 5, Table S1). Within Dinophyceae (0.08–2.45%), photosynthetic forms, such as *Heterocapsa*, *Karlodinium*, *Prorocentrum* and *Protodinium*, and heterotrophic forms, including *Gyrodinium* and *Warnowia,* were detected along the water column. Syndiniales (0.47–23.1%), mainly endoparasites, were distributed along the water column, with higher abundances at the lower end of the oxycline of both stations. Representatives of the five main Syndiniales groups (I–V) were detected, with Groups I (up to 6.4%) and II (up to 16.2%) as the most abundant. Within Group I, Clade 1 had the highest relative abundance (up to 6.2%), mainly at the lower end of the oxycline of both stations, while Clades 2 (up to 2.2%) and 5 (up to 1.6%) were well represented at the oxycline of St2. Group II comprised diverse clades, with the highest relative abundances occurring at the oxycline of St5.

Large heterotrophic groups (>3 μm) such as Ciliophora (up to 0.9%; represented by Spirotrichea) and Radiolaria (up to 83.7%) were also found in the study area (Figs 4 and 5, Table S1). Radiolaria dominated the oxycline of the slope station St2 and was mainly represented by Spumellaria, such as *Cladococcus* (77%), *Heliosphaera* (4.2%) and *Astrosphaera* (1.1%). Finally, Sagenista, heterotrophic uncultured pico- and nanoflagellates, were especially well represented at the oxycline of the oceanic station St5 by MAST-4B (1.3%), MAST-4C (3.6%), MAST-4E (0.52%), MAST-7B (1.1%), MAST-9A (0.6%) and MAST-11 (2.3%) (Fig. 5, Table S1).

### Diversity and structure of the eukaryotic picoplankton

**Fig. 6:**
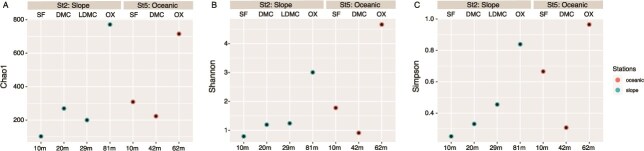
Comparison of picoeukaryotic alpha diversity indices between the two sampling stations at different depths: SF, DCM, LDCM and OX.

Diversity indices showed a high variation among the two stations and the sampling depths (Fig. 6). The highest values of these indices were found at the lower end of the oxycline of both stations, whereas the lowest values were found in the surface waters (10 and 20 m depth) of the slope station St2 and at the DCM layer of the oceanic station St5.

**Fig. 7:**
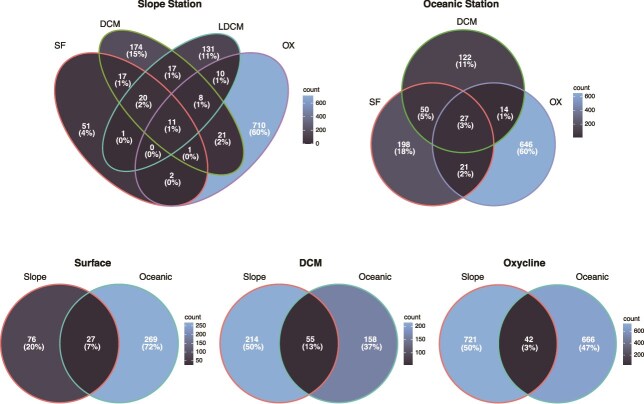
Venn diagram showing the numbers of shared and unique ASVs within each station (slope and oceanic stations) and between the two stations by sampling depths: SF, DCM and OX. Labels for sampling depths: SF, surface; DCM, deep chlorophyll maximum; LDCM, lower limit of the DCM in the slope station; OX, end of the oxycline.

Along the water column, the highest numbers of unique ASVs were found at the oxycline of both stations (721 ASVs at St2 and 666 ASVs at St5, belonging to 108 and 127 genera, respectively) (Figs 7 and S8). At the surface, the highest numbers of unique ASVs were detected at the oceanic station St5 (269 ASVs, belonging to 95 genera), whereas, at the DCM, the highest numbers of unique ASVs were detected at the slope station St2 (214 ASVs, belonging to 52 genera). In addition, almost exclusive communities were found at each studied sample in both stations, since they only shared 7, 13 and 3% ASVs at the surface, DCM and oxycline, respectively (Fig. 7). These results reflect the differences in the distribution of the picoplanktonic communities within each station, which shared only 1% (St2) and 3% (St5) of ASVs among all the sampling depths, with the oxycline having the highest number of unique ASVs along the water column at each station (60% ASVs belonging to 105 genera at St2 and 116 genera at St5) (Figs 7 and S8).

The PCoA analysis showed three groups of picoeukaryotic communities: one at the surface and subsurface (DCM and LDCM) layers of both stations and two different communities at the lower end of the oxycline of each station (Fig. S9). This trend was confirmed by PERMANOVA (Table S3) and ANOSIM (Table S4) analyses. The communities of the surface and subsurface layers were related to high oxygen concentration (*P* < 0.1), whereas at the lower end of the oxycline, the community of the slope station St2 was positively related to salinity (*P* < 0.01), SiO_2_ (*P* < 0.05) and dissolved inorganic N (*P* < 0.1), and the community of the oceanic station St5 was positively associated to PO_4_^−3^ (*P* < 0.1) (Fig. S9, Table S5).

## DISCUSSION

### Oceanographic conditions and picoplankton abundances

The ETNP in the Mexican Pacific hosts the largest OMZ in the global ocean (Paulmier and Ruiz-Pino, 2009), encompassing highly productive areas with complex climatic and oceanographic processes (Ito and Deutsch, 2013). The information derived from satellite images, particularly SST, Kd490 and chlorophyll *a* (Fig. S4), as well as the CUI index (Fig. S3) and the hydrographic data from the studied transect in this OMZ (Fig. S5) indicate a weak upwelling event (post-upwelling) outlined by the ascend of cooler water (21°C isotherm at 30 m depth) close to the coastal station St1. Furthermore, a strong upwelling event prior to the cruise (4 April 2018; Fig. S3) may explain the shoaling of isotherms near the coast (St1 and St2).

These upwelling events enhanced the transport of cold, nutrient-rich, low-oxygen waters (StSsW) to the surface and drove the warm, oxygenated TSW mass from the coastal station St1 (within Acapulco Bay) offshore (Figs S1, S2 and S5). This coastal displacement of the TSW and the ascent of the deeper StSsW to the surface could partially explain the lower abundance of picoeukaryotes, detected by flow cytometry, at the slope station (St2) and their increase at the oceanic station (St5) (Fig. 3). This is also supported by the relatively high concentrations of NO_3_^−^, PO_4_^−3^ and SiO_2_ at the surface of the most coastal station (St1) (Figs 2 and S6). The arrival of StSsW to the coast caused the upper limit of the OMZ (9 μM O_2_) to rise to 50 m depth from the intermediate station St3 to the most oceanic station St5 (Fig. S5), similar to previous studies in the tropical Mexican Pacific (Cepeda-Morales *et al*., 2013; Trucco-Pignata *et al*., 2019; Pajares *et al*., 2020).

Subsurface thermoclines and DCM peaks were observed to deepen from the coast towards the open ocean, along with oxyclines associated with the thermoclines (Fig. S5). In addition, an intense second DCM was detected only at the oceanic station St4, a typical condition of remote areas in the Mexican Pacific OMZ (Cepeda-Morales *et al*., 2009; Márquez-Artavia *et al*., 2019). Nutrient profiles showed a similar pattern along the transect, increasing with depth and displaying characteristic nutriclines, except for NH_4_^+^, with a surface maximum, and NO_2_^−^, which peaked at the oxycline. These distinctive patterns, influenced by the upwelling condition and previously reported for the study area (Hernández-Becerril *et al*., 2018, 2024; Santana-Vega *et al*., 2018; Pajares *et al*., 2020), likely contributed to the observed differences in the diversity, abundance and distribution of picoeukaryotic communities.

The abundances of both prokaryotic and eukaryotic picoplankton obtained by flow cytometry exclusively represent photosynthetic forms; thus, heterotrophic forms, such as Syndiniales, Radiolaria, Ciliophora or Sagenista, were not quantified. The larger contribution of eukaryotic picoplankton (67.7%) to the total picophytoplankton biomass (8.5 μg C l^−1^) compared to the prokaryotic picoplankton (*Synechococcus* and *Prochlorococcus*) was unsurprising, given the larger size of eukaryotic cells. Furthermore, these contributions were closely linked to chlorophyll *a* concentrations since only the photosynthetic forms were considered. Due to the high nutrient requirements, picoeukaryotes typically form surface maxima when upwelling provides cooler, nutrient-rich waters (Jiao *et al*., 2002; Marty *et al*., 2008). This is consistent with this study, where the highest abundance of picoeukaryotes coincided with the DMC at St5, a layer in which they can contribute up to 50% of the total chlorophyll *a* (Not *et al*., 2008; Cabello *et al*., 2016; Gérikas-Ribeiro *et al*., 2016).

### Distribution and diversity of the eukaryotic picoplankton

Differential vertical and horizontal distributions of picoeukaryotes were observed in the sampled stations with some abundant taxonomic groups only found in specific samples (Figs 4 and 5). For instance, consistent with previous findings in the study area (e.g. high picoeukaryote abundance from flow cytometry and photosynthetic pigment analysis by High Performance Liquid Chromatography -HPLC-, with considerable concentrations of chlorophyll *b* and prasinoxanthin; Hernández-Becerril *et al*., 2024), 18S rRNA sequencing revealed high relative abundance and diversity of chlorophytes, particularly Mamiellophyceae, across all samples. Key contributors in this ETNP region included *Ostreococcus* sp. and *Bathycoccus prasinos*, which dominated the surface and subsurface layers (presumably well-illuminated layers), and *Micromonas commoda*, which was prominent at the lower end of the oxycline of the oceanic station St5. Mamiellophyceae were also found in high relative abundance in surface and DCM layers during the Tara Ocean expedition (Lopes dos Santos *et al*., 2017a) and in the OMZ waters off central Chile (de la Iglesia *et al*., 2020). As globally abundant primary producers, these photosynthetic picoeukaryotes play a significant role in enhancing oceanic diversity and biomass (Worden, 2006; Vaulot *et al*., 2008; Worden and Not, 2008; Tragin and Vaulot, 2019).

A highly abundant group of picoplanktonic chlorophytes, detected for the first time in the Mexican Pacific, was the recently described Chloropicophyceae (Lopes dos Santos *et al*., 2017b). Their global significance in surface waters was highlighted during the Tara Ocean expedition (Lopes dos Santos *et al*., 2017a) and specifically in tropical and subtropical waters of the Atlantic and Pacific Oceans (Charvet *et al*., 2021; Lin *et al*., 2022). The two described genera, *Chloroparvula* and *Chloropicon,* were revealed by metabarcoding in this study, with *Chloropicon* being particularly abundant at the lower end of the oxycline of the slope station St2.

The occurrence of Prasinodermophyta, another recently described group represented by *P. coloniale* (Li *et al*., 2020), was detected in relative low abundance exclusively at the lower end of the oxycline of the oceanic station St5. Previous unreported results from microscopic observations in subsurface layers of this study area revealed highly abundant populations of a spherical species closely resembling *P. coloniale*. Therefore, this study might identify the dense populations observed in earlier analyses.

Within Ochrophytes, heterotroph flagellates such as *Spumella,* an active predator of prokaryotes and other picoeukaryotes, were relative abundant at the deeper part of the oxyclines at both stations. Additionally, photosynthetic Chrysophyceae (Clades H and G), Dictyochophyceae (Florenciellales and Pedinellales) and Parmales (Bolidophyceae with siliceous shileds) were prevalent at the lower end of the oxycline at St5. They are well-known picoplanktonic groups with a cosmopolitan distribution (Díez *et al*., 2001; Vaulot *et al*., 2008; Fujita and Jordan, 2017), but most of them are reported here for the first time at the oxycline of an OMZ. Notably, Parmales have previously been detected by SEM in subsurface layers of Mexican waters close to the study area (Bravo-Sierra and Hernández-Becerril, 2003).

Diatoms often form dense blooms in upwelling regions, contributing significantly to nano- and microplankton communities and serving as key components of eukaryotic assemblages in oxygen-depleted waters (Montero *et al*., 2007; Wang *et al*., 2017). Occasional blooms and high densities of diatoms, such as *Chaetoceros*, *Leptocylindrus* and *Pseudo-nizschia*, have been reported in locations near the study area (Hernández-Becerril *et al*., 2024). However, the low relative abundance of pico-diatoms observed in this study, primarily detected at the slope station St2, may be attributed to the absence of blooms during the sampling time and that most diatoms are larger in size. This finding is consistent with a previous report from the OMZ off Chile (Parris *et al*., 2014).

On the other hand, the detection of Prymnesiophyceae (Haptophyte) and Chlorarachniophyceae (Cercozoa) in low relative abundances, primary at the lower end of the oxycline of St5, aligns with previous reports of Prymnesiophyceae with picoplankton representatives (Moon-van der Staay *et al*., 2001; Vaulot *et al*., 2008; Acosta *et al*., 2013), and the global distribution of chlorarachniophytes (Vaulot *et al*., 2008).

The most abundant groups of Syndiniales identified in this study, particularly at the lower end of both oxyclines, were Groups I and II, which include clades predominantly retrieved from anoxic and suboxic environments (Guillou *et al*., 2008; de la Iglesia *et al*., 2020). Syndiniales are parasites with a life cycle of <3 days, resulting in the release of hundreds of free-living dispersive dinospores belonging to the picoplankton size fraction (Guillou *et al*., 2008). Therefore, these environmental sequences likely result from such dinospores. These findings are highly coincident with previous studies in the region, which reported the presence of these groups in the 1.6–30 μm fraction but in deeper waters (800 m depth within the OMZ core and 1 000 m depth at the lower oxycline) (Duret *et al*., 2015). Furthermore, Syndiniales dominated the 0.2–1.6 μm fraction in the OMZ off northern Chile, exhibiting depth-specific variation in composition and total richness despite uniform oxygen conditions (Parris *et al*., 2014). A proposed explanation involves microniches on the surface of sinking particles (Duret *et al*., 2015; Bertagnolli and Stewart, 2018). Additionally, another study in the OMZ off Costa Rica indicated a shift from photosynthetic dinoflagellates at the surface to parasitic dinoflagellates and ciliates in deeper waters, suggesting selective pressure on protist communities (Jing *et al*., 2015).

Among heterotrophic protists, the significant relative abundance of various Sagenista groups belonging to MAST (4, 7, 9, 11) at the lower end of the oxycline at the oceanic station St5, particularly MAST-4, supports the hypothesis that these groups play a key role as bacterial grazers (Massana *et al*., 2004; Massana and Pedrós-Alió, 2008; Lin *et al*., 2012; Latorre *et al*., 2021; Rodríguez-Martínez *et al*., 2022). This observation also aligns with previous studies reporting MAST-9 as the most abundant MAST group in anoxic marine environments (Massana *et al*., 2014; Kang and Kang, 2023). Moreover, MAST-4 and MAST-7 have been reported in OMZ regions (Giner *et al*., 2020).

Additionally, significant amounts of larger taxa (>2–3 μm) belonging to Radiolaria (mostly *Cladococcus*) and Ciliophora (Spirotrichea) were found in the study area, primarily at the lower end of the oxycline of St2, similar to observations from other regions of the global ocean (Not *et al*., 2007; de Vargas *et al*., 2015; Li and Endo, 2020), and particularly in suboxic–anoxic layers (Orsi *et al*., 2012; Parris *et al*., 2014; de la Iglesia *et al*., 2020; Suter *et al*., 2022). Several hypotheses have been proposed to explain the notable abundance of these protists within picoeukaryotic communities, including that “pico-sized radiolarians” represent gametes of normal-sized radiolarians (Massana, 2011; Li and Endo, 2020). Moreover, sequences derived from supposedly larger cells might result from a combination of filtration artifacts (broken cells) and the amplification of detrital DNA (Not *et al*., 2007; Giner *et al*., 2020).

Finally, a relative low abundance of ASVs remained unassigned to any phylum or supergroup (up to 1.5%, Fig. 4), with the highest representation in the oceanic oxycline, in which a high relative abundance of ASV87 was observed (Table S1). A BLAST analysis of this ASV showed 99.2% identity (with 100% query cover; Table S2) to a sequence from the Northeastern Red Sea (dissolved oxygen of 4.25 mg l^−1^) assigned to uncultured stramenopiles (Acosta *et al*., 2013). This finding, along with the high abundance of ASVs unassigned at the genus level, most of which were from the lower end of the oxyclines at both stations (Table S1), indicates that many microorganisms in these oxycline ecosystems remain understudied.

Overall, a strong stratification of picoeukaryotic communities was observed in the study area, consistent with findings from other parts of the global ocean (Not *et al*., 2007; Cabello *et al*., 2016; Giner *et al*., 2020), particularly in oxygen-depleted waters (e.g. ﻿ Schnetzer *et al*., 2011; Orsi *et al*., 2012; de la Iglesia *et al*., 2020). Furthermore, picoeukaryotic diversity increased with depth, which is in line with other reports from the OMZ off Chile and Costa Rica (Parris *et al*., 2014; Jing *et al*., 2015) and mirroring diversity patterns observed in prokaryotic communities in OMZ areas close to the study area (Pajares *et al*., 2020, 2023). The highest diversity and unique picoeukaryotic taxa at the lower end of the oxycline layer of both stations strongly suggest that oxygen concentration is a crucial driver of microbial community distribution in this OMZ (Fenchel and Finlay, 2008; Bertagnolli and Stewart, 2018), especially the picoeukaryotic community. Oxyclines likely provide diverse niches that support the growth of different trophic groups (photosynthetic organisms, heterotrophs, mixotrophs and parasites), thereby promoting hotspots of metabolic activity (Edgcomb, 2016; Giner *et al*., 2020). Along with sparse oxygen concentrations, factors such as sinking organic matter and high nutrient availability during the upwelling season further contribute to the high microbial diversity within oxyclines (Bertagnolli and Stewart, 2018; Pajares *et al*., 2023).

## CONCLUSIONS

This study reveals the high diversity and spatial heterogeneity of picoeukaryotic communities in the tropical Mexican Pacific OMZ. Photosynthetic groups, such as Chlorophyta and Ochrophyta, along with non-photosynthetic groups such as Syndiniales, Sagenista and Radiolaria were prominent. The first report of Chloropicophyceae and Prasinodermophyta in this region offers new insights into its picoeukaryotic diversity.

Oxygen availability and post-upwelling conditions were key in shaping community structure. Photosynthetic picoeukaryotes were less abundant at the slope station than at the oceanic station. Furthermore, substantially different communities were found at the lower end of the oxyclines of both stations. Almost exclusive communities were found in all depths, with photosynthetic organisms (mostly Mamiellophyceae) dominating surface and subsurface layers, whereas mixotrophic, heterotrophic and parasitic forms prevailed at both oxyclines, where the highest diversity was found. These results underscore the crucial influence of oxygen in structuring picoplankton communities and identify oxyclines as hotspots of specialized niches that favour high picoplankton diversity and trophic strategies, with important implications for understanding microbial ecology in oxygen-depleted environments.

## Supplementary Material

SUPPLEMENTARY_FIGURES_fbae083

TableS1_RQ_fbae083

SUPPLEMENTARY_TABLES_fbae083

## Data Availability

Raw sequence data were deposited in the NCBI Sequence Read Archive under the BioProject accession number PRJNA1090064.
