# Supplementary material for: Diversity and distribution of the eukaryotic picoplankton in the oxygen minimum zone of the tropical Mexican Pacific
Source: J Plankton Res. 2025 Mar 1;47(2):fbae083. doi: 10.1093/plankt/fbae083 (PMC11879187; doi:10.1093/plankt/fbae083)

**SUPPLEMENTARY FIGURES**

**Figure S1**. Cytograms of scatter and fluorescence obtained from one of the samples collected along the Acapulco’s transect. *Prochlorococcus* (blue), *Synechococcus* (green and yellow), and picoeukaryotes (red) are discriminated based on the fluorescence of their natural pigments: chlorophyll (red) or phycoerythrin (orange). 2.0 μm beads (black) were added as internal reference:

A) Red fluorescence *vs* orange fluorescence; B) Side scatter (which provide information about the size of the cell and refractive properties of the cell) *vs* red fluorescence.

**Figure S2**. Quarterly plots of temperature Θ (ºC) - salinity *S_A_* (g kg^-1^) diagrams for all sampling stations along the Acapulco’s transect during the cruise (physicochemical data were obtained down to 1200 m depth at the most oceanic stations). The lines represent the limits of the water masses: Tropical Surface water (*TSW*), Transitional water (*TrW*), Subtropical Subsurface water (*StSsW*), and Pacific Intermediate water (*PIW*).


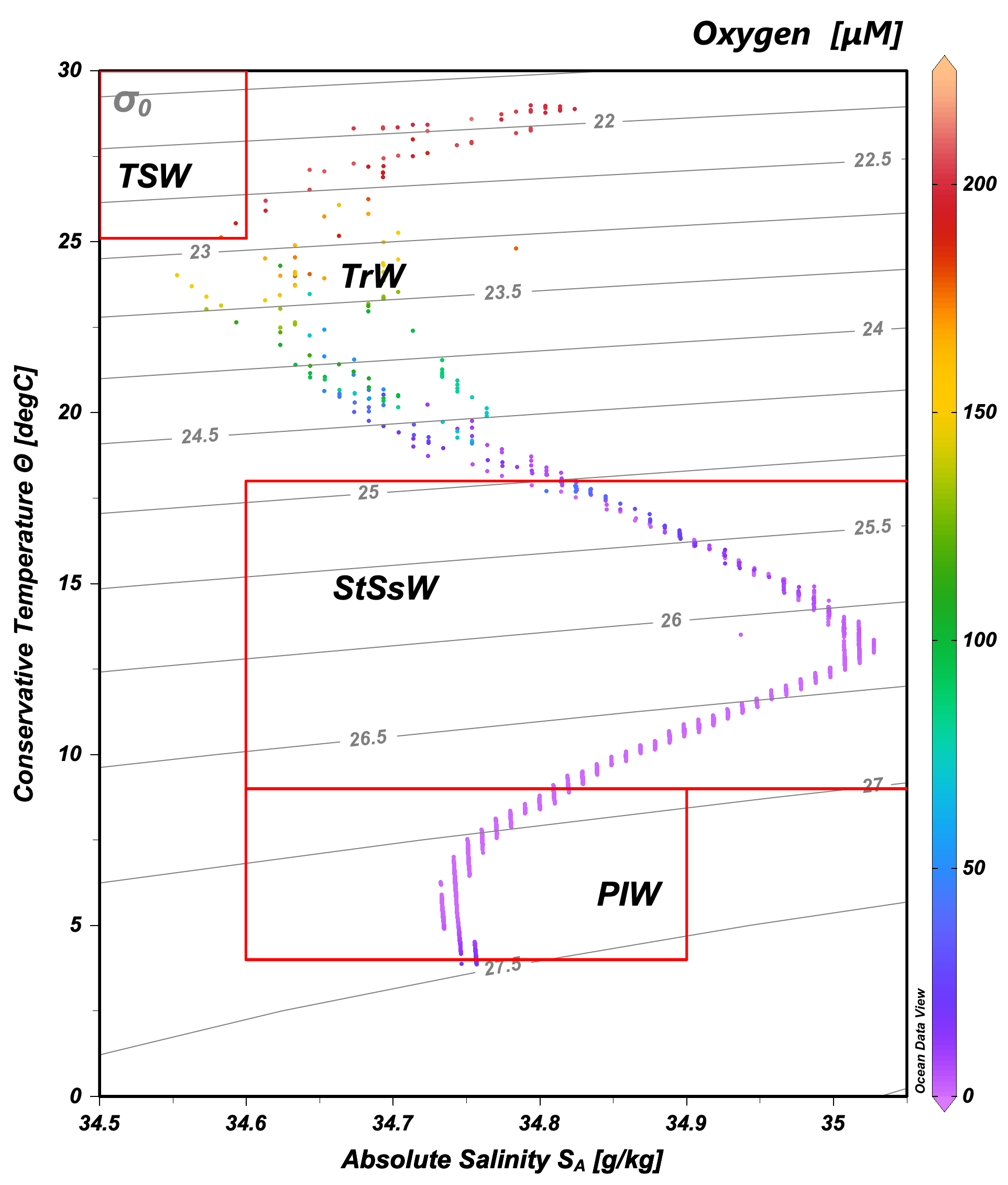


**Figure S3**. Daily variation of the Coastal Upwelling Index (CUI) for April 2018. The red rectangle highlights the study period.


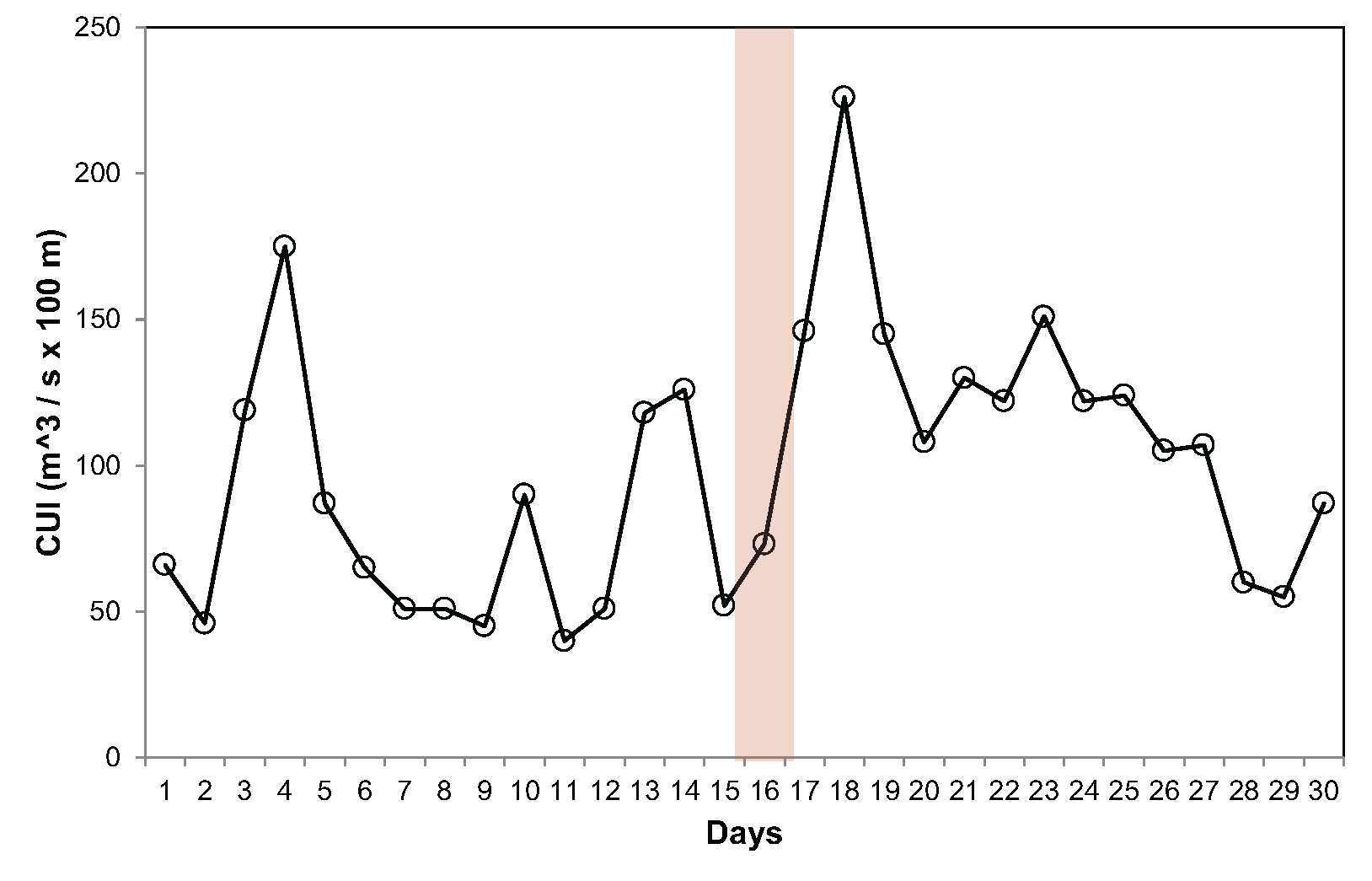


**Figure S4**. High-resolution satellite imageries of oceanographic data of the Tropical Mexican Pacific during the cruise obtained from MODIS-Aqua.

****a) Mean sea surface temperature (SST).

b) Diffuse attenuation coefficient at 490 nm (Kd490).

c) Mean surface chlorophyll-*a*.

**Figure S5**. Vertical distribution of physico-chemical variables (dissolved oxygen, chlorophyll-a, salinity, and temperature) along the Acapulco’s transect (five stations) during the cruise.

**Figure S6**. Vertical distribution of nutrient concentrations along the Acapulco’s transect (five stations) during the cruise: ammonium (NH_4_^+^), nitrate (NO_3_^–^), soluble reactive phosphorus (PO_4_^–3^), and soluble reactive silica (SiO_2_).


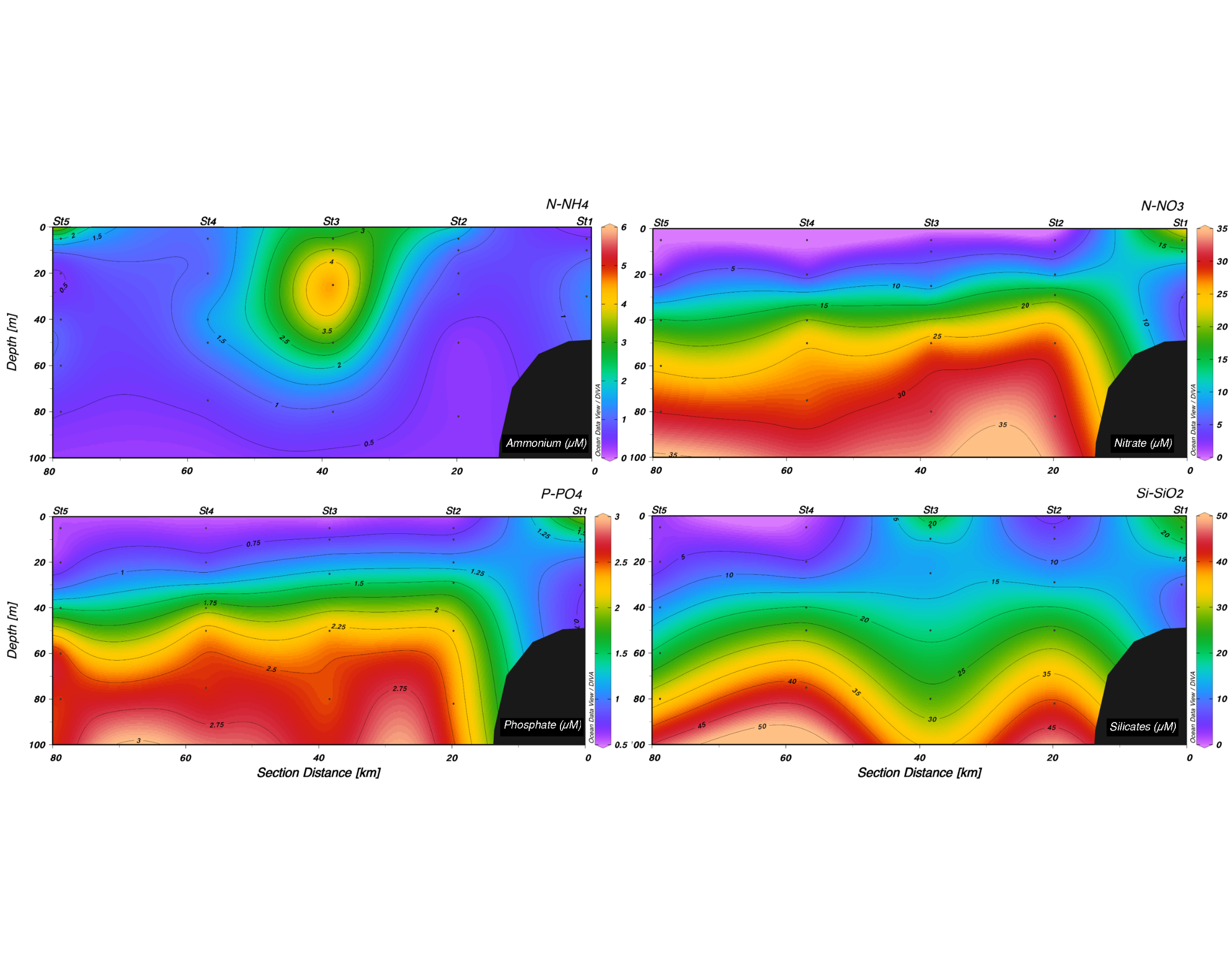


**Figure S7.** Scanning Electron Microscopy (SEM) micrographs of representative siliceous picoeukaryotes found in the two sampling stations: A) *Thalassiosira oceanica*, B) *Minidiscus chilensis*; and C) *Triparma laevis*.

**
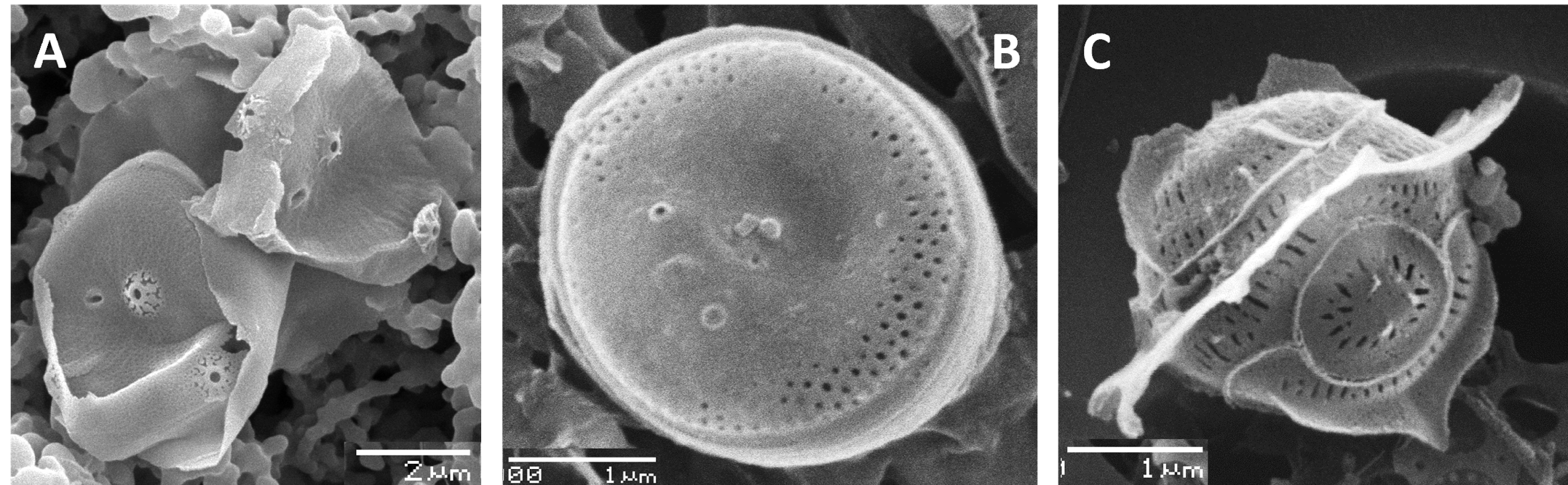
**

**
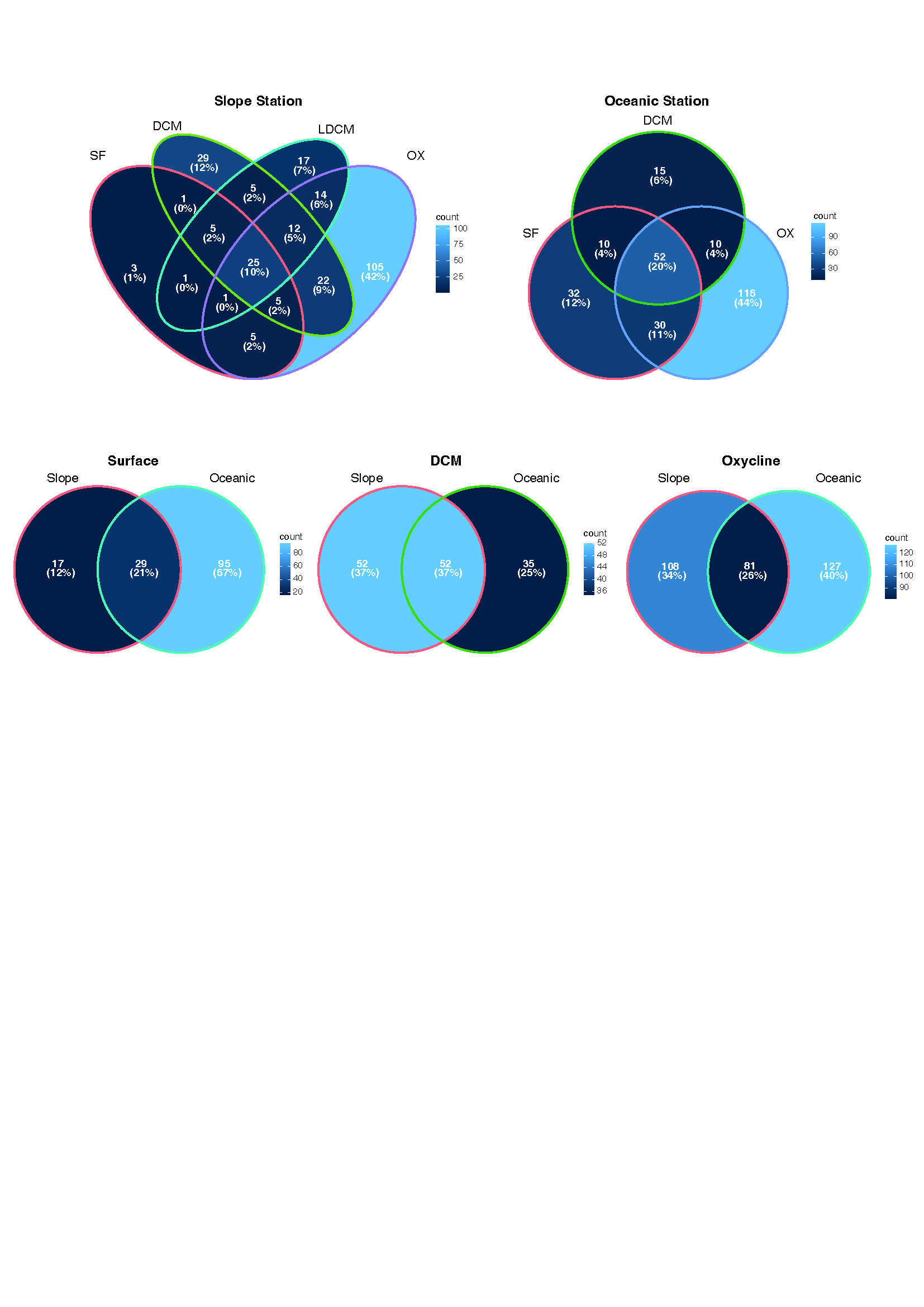
Figure S8**. Venn diagrams showing the numbers of shared and unique taxa at genus level withing each station (Slope and Oceanic station) and between the two stations by sampling depths: surface, DCM and oxycline. Labels for sampling depths: SF, surface; DCM, deep chlorophyll maximum; LDCM, lower limit of the DCM in the slope station and OX, oxycline.

**Figure S9**. Plot of principal coordinate analysis (PCoA) of the samples based on a Bray-Curtis distance matrix of ASVs. Symbols: shapes, stations; fill colors, sampling depths. Length and angle of arrows: extend of correlation between significant environmental variables (p < 0.05 and p < 0.1, dashed lines) and PCoA axes. SF, surface; DCM, deep chlorophyll maximum; LDCM, lower limit of the DCM in the slope station; OX, oxycline; DIN, dissolved inorganic nitrogen.


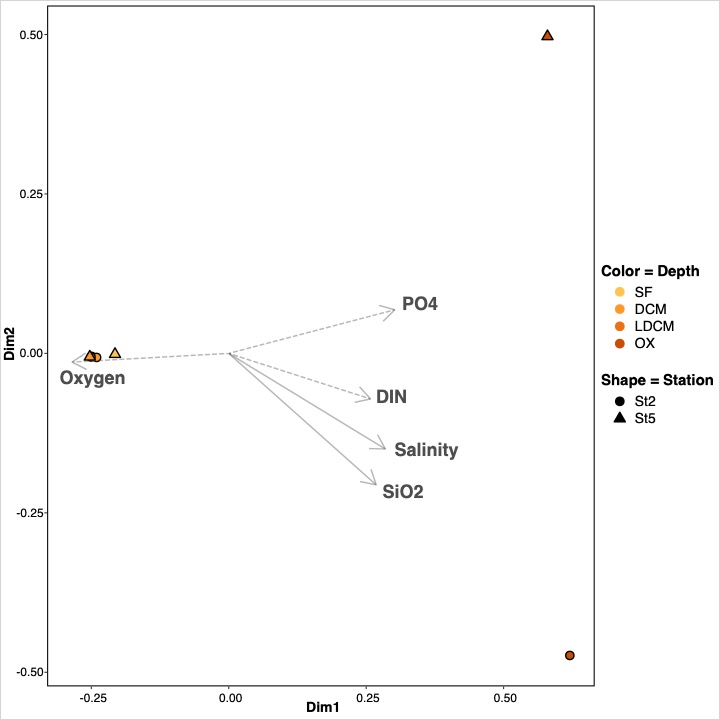

Supplement: SUPPLEMENTARY_FIGURES_fbae083 [file supplementary_figures_fbae083.docx]
