# Supplementary material for: Diversity and distribution of the eukaryotic picoplankton in the oxygen minimum zone of the tropical Mexican Pacific
Source: J Plankton Res. 2025 Mar 1;47(2):fbae083. doi: 10.1093/plankt/fbae083 (PMC11879187; doi:10.1093/plankt/fbae083)
Supplement: SUPPLEMENTARY_TABLES_fbae083 [file supplementary_tables_fbae083.docx]

**SUPPLEMENTARY TABLES**

**Table S1**. Final ASV table from DADA2 analysis showing taxonomy classification, relative abundances (%), and read counts per sample (see excel file).

**Table S2.** Key picoeukaryotic components (from Division to species) identify in this study showing high similarity based on Blast analysis.

**Division/Phylum Class/Order/Group Genus/Clade Species ASV # Blast similarity NCBI Accession n°**

Chlorophyta Mamiellophyceae *Ostreococcus* *O.* sp. clade B 1 100% EU143476

*O*. *lucimarinus* 61 100% KT860897

*Micromonas* *M*. *commoda* 34 99.2% LC806245

*Bathycoccus* *B*. *prasinos* 6 100% MT571465

Chloropicophyceae *Chloropicon* *C*. *roscoffensis* 26 100% MT489379

*C*. *sieburthii* 574 100% KU843585

*Chloroparvula* *C*. *pacifica* 17 98.9% KU843574

Prasinodermophyta Prasinodermophyceae *Prasinoderma* *P*. *coloniale* 1384 99.7% KT860885

Ochrophyta Chrysophyceae *Spumella* *S. oblata* 56 99.4% MZ420288

1195 100% KF129656

Clade H 1181 100% KF130056

Dictyophyceae *Florenciella* *F*. *parvula* 1240 100% LC779584

Pelagophyceae *Pelagomonas* *P*. *calceolata* 23 100% EF455763

Bolidophyceae *Triparma* *T*. *eleuthera* 388 100% KR998400 *T*. *pacifica* 1226 100% HQ912557

Bacillariophyceae *Thalassiosira* *T*. *oceanica* 467 96.3% KY980420

*Chaetoceros* *C*. *tenuissimus* 720 100% MG972315

*Minidiscus* 140 100% MH843669

Haptophyta Prymnesiophyceae *Chrysocromulina* 241 100% MZ611704

*Phaeocystis* *P*. *globosa* 864 100% MN826692

Cercozoa Chlorarachniophyceae *Chlorarachnion* *C*. *reptans* 1363 98.6% U03477

*Partenskyella* *P*. *glossopodia* 1267 100% KF422630

*Minorisa* *M*. *minuta* 1304 100% LC375245

Dinoflagellata Dinophyceae *Heterocapsa* *H. rotundata* 160 99.2% KY980277

*Karlodinium* *K. veneficum* 1806 100% EF036540

*Protodinium P. simplex* 1934 99.7% U41086

*Gyrodinium* *G. fusiforme* 114 100% AB120002

*Warnowia* 104 99.7% FJ914412

Syndiniales Group I Clade 1 31 100% EU793901

Group I Clade 1 346 100% HQ865186

Group I Clade 2 472 99.7% GU819954

Group I Clade 5 49 99.7% EU793849

Group II Clade 1 146 100% (*Amoebophrya*) MT269120

Radiolaria Polycystinea *Cladococcus* *C*. *scoparius* 264 100% KJ759943

*C*. *scoparius* 468 100% EF172899

*Heliosphaera* 474 100% KP175030

*Astrophaera A. hexagonalis* 487 100% GU821297

Ciliophora Spirotrichea *Pseudotontonia*  53 99.7% MT973830

Sagenista MAST-4B 1187 100% MW521832

MAST-4C 1183 100% MW521833

MAST-4E 1214 99.7% MW521834

MAST-7B 1205 97.3% MW521835

MAST-9A 1224 100% MW521837

MAST-11 1189 98.4% MW521838

Unassigned NA 87 99.2% (*Stramenopiles*) KC583028

- Red text indicates tentative taxonomic assignments based on BLAST analysis of unassigned groups using the PR2 database. NA: not assigned.

**Table S3.** Permutational multivariate analyses of variance (PERMANOVA) between the two stations and depths for ASV community structure using the Bray-Curtis distance matrix with 5039 permutations.

| **Metrics** | **Df** | **Sums Sqs** | **R^2^** | **F** | **Pr (>F)** |
| --- | --- | --- | --- | --- | --- |
| Stations | 1 | 0.17713 | 0.11036 | 0.6202 | 0.574 |
| Residual | 5 | 1.42793 | 0.88964 |  |  |
| Total | 6 | 1.60506 | 1.00000 |  |  |
| Depths | 1 | 1.00868 | 0.62826 | 8.4467 | 0.049 |
| Residual | 5 | 0.59708 | 0.37184 |  |  |
| Total | 6 | 1.60576 | 1.00000 |  |  |

**Table S4.** Analyses of similarity (ANOSIM) between the two stations and depths for ASV community structure using the Bray-Curtis distance matrix with 5039 permutations.

| **Metrics** | **R statistic** | **p-value** |
| --- | --- | --- |
| Stations | -0.07407 | 0.601 |
| Depths | 0.9818 | 0.042 |

**Table S5.** Results of the statistical analysis (p-values) of the principal coordinate analysis (PCoA) for the two ordination scores related to the environmental variables.

| **Variables** | **Dim1** | **Dim2** | **R^2^** | **Pr (>r)** | **Sign** |
| --- | --- | --- | --- | --- | --- |
| PO_4_ | 0.97529 | 0.22091 | 0.7679 | 0.05195 | **.** |
| SiO_2_ | 0.79285 | -0.60941 | 0.9158 | 0.04795 | * |
| DIN | 0.96375 | -0.26681 | 0.5731 | 0.05694 | **.** |
| Conductivity | -0.95577 | 0.29411 | 0.5452 | 0.14386 |  |
| Density | 0.95874 | -0.28429 | 0.5866 | 0.14386 |  |
| Fluorescence | -0.96664 | 0.25613 | 0.1773 | 0.38761 |  |
| Oxygen | -0.99886 | -0.04767 | 0.6521 | 0.09590 | **.** |
| Salinity | 0.88490 | -0.46579 | 0.8297 | 0.00999 | ** |
| Temperature | -0.95323 | 0.30224 | 0.5853 | 0.14386 |  |

Significance: “**”, 0.01; “*”, 0.05; “**.**”, 0.1. P values based on 5039 permutations.
